# Genomic consequences of apple improvement

**DOI:** 10.1038/s41438-020-00441-7

**Published:** 2021-01-01

**Authors:** Zoë Migicovsky, Kyle M. Gardner, Christopher Richards, C. Thomas Chao, Heidi R. Schwaninger, Gennaro Fazio, Gan-Yuan Zhong, Sean Myles

**Affiliations:** 1 Department of Plant, Food, and Environmental Sciences, Faculty of Agriculture, Dalhousie University, Truro, NS Canada; 2 USDA-ARS, Plant Germplasm Preservation Research Unit, Fort Collins, CO USA; 3 USDA-ARS, Grape Genetics Research Unit, Geneva, NY USA; 4 Present Address: Agriculture and Agri-Food Canada, Fredericton Research and Development Centre, Fredericton, NB Canada

**Keywords:** Agricultural genetics, Population genetics

## Abstract

The apple (*Malus domestica*) is one of the world’s most commercially important perennial crops and its improvement has been the focus of human effort for thousands of years. Here, we genetically characterise over 1000 apple accessions from the United States Department of Agriculture (USDA) germplasm collection using over 30,000 single-nucleotide polymorphisms (SNPs). We confirm the close genetic relationship between modern apple cultivars and their primary progenitor species, *Malus sieversii* from Central Asia, and find that cider apples derive more of their ancestry from the European crabapple, *Malus sylvestris*, than do dessert apples. We determine that most of the USDA collection is a large complex pedigree: over half of the collection is interconnected by a series of first-degree relationships. In addition, 15% of the accessions have a first-degree relationship with one of the top 8 cultivars produced in the USA. With the exception of ‘Honeycrisp’, the top 8 cultivars are interconnected to each other via pedigree relationships. The cultivars ‘Golden Delicious’ and ‘Red Delicious’ were found to have over 60 first-degree relatives, consistent with their repeated use by apple breeders. We detected a signature of intense selection for red skin and provide evidence that breeders also selected for increased firmness. Our results suggest that Americans are eating apples largely from a single family tree and that the apple’s future improvement will benefit from increased exploitation of its tremendous natural genetic diversity.

## Introduction

Plant domestication is the artificial selection for phenotypic changes. These changes are often maladaptive to the organism in the wild, but of benefit to humans, leading to genetic differentiation between the wild progenitor species and contemporary commercial cultivars. In many species, domestication involves an intense population bottleneck, which reduces genetic variation. In contrast, long-lived perennial species, including fruit trees, generally experienced mild domestication bottlenecks owing to several factors including the extensive use of clonal propagation, long juvenile phases, and predominantly outcrossing mating habit^1^.

The domesticated apple (*Malus* x *domestica* Borkh.), is one of the most economically important fruit crops, with a worldwide production value exceeded only by tomatoes (*Solanum lycopersicum* L.) and grapes (*Vitis vinifera* L.)^2^. In the first half of the twentieth century, Nikolai Vavilov suggested that the origin of apple domestication coincided with the centre of phenotypic diversity of the presumed ancestral species *Malus sieversii* (Ledeb.) Roem., located in the Tien Shan mountains along Kazakhstan’s eastern border with China^3^. This speculation was largely supported by recent analyses of genetic data, which identified *M. sieversii* as the primary progenitor species for *M. domestica*^4^. In addition, over 20% of the *M. domestica* genome was likely derived from the wild European crabapple *Malus sylvestris* (L.) Mill^5,6^.

Plant domestication can be understood as a continuum rather than a single event, and selection continues over time as plant breeders target traits for improvement. While there is an immense amount of genetic variation available in apple due to a weak domestication bottleneck^6,7^, most commercial production focuses on a limited number of elite cultivars. In 2017, over 50% of the commercial apple production in the European Union consisted of only five apple cultivars^8^. The repeated use of a small number of elite cultivars during breeding reduces genetic diversity, especially among commercial cultivars. In addition, clonal propagation allows successful cultivars to persist for long periods of time, such as the ‘McIntosh’ apple, which is still in widespread production after 200 years^9,10^. The ability of the apple industry to respond to pests, pathogens, and a changing climate will rely on comprehensive evaluations of apple variation, and the subsequent introgression of desirable genetic variants into apple breeding material.

In this study, we investigated (1) the domestication history of *M. domestica* by examining its relationship to the progenitor species *M. sieversii* and *M. sylvestris*, (2) signatures of positive selection during domestication and improvement, and (3) the improvement history of *M. domestica* by examining the relationships among modern cultivars in the USDA apple germplasm collection.

## Materials and methods

### Genotype data collection

The apples (*Malus* spp.) investigated here are from the USDA apple germplasm repository in Geneva, NY, USA. Leaf tissue was collected from 1949 accessions. The countries of origin of the accessions are indicated in Supplementary Fig. S1 and Supplementary Table S1. DNA was extracted from these accessions using commercial extraction kits. Genotyping-by-sequencing (GBS) libraries were generated according to Elshire et al.^11^. A visual overview of all data processing and analysis steps described below is provided in Supplementary Fig. S2.

The samples were processed with two different restriction enzymes (ApeKI, PstI/EcoT22I) in separate GBS libraries and were sequenced using Illumina Hi-Seq 2000 technology (96 samples per lane) at Cornell University (Ithaca, New York, US) across 42 lanes generating 100-bp single-end reads. The DNA sequence data are as NCBI BioProject PRJNA636391. Reads that failed Illumina’s ‘chastity filter’ were removed and remaining reads were aligned to the *Malus* x *domestica* GDDH13 v1.1 reference genome^12^ using Burrows-Wheeler aligner tool v0.7.12^13^ and the Tassel version 5 pipeline^14^. Kmerlength was set to 82 for ApeKI and 89 for PstI-EcoT22I, and the minMAF was set to 0.01 during the DiscoverySNPCallerPluginV2 step. Non-biallelic sites and indels were removed using VCFtools v0.1.14^15^. VCF files for both enzymes were then merged using a custom Perl script that preferentially kept SNPs called from the PstI-EcoT22I libraries in cases where SNPs were identified from both restriction enzymes. The resulting data set contained 1949 accessions and 1,103,605 SNPs. Mean read depth per individual, per SNP, and the proportion of heterozygotes per site were calculated using VCFtools v0.1.14^15^ (Supplementary Fig. S3).

Missing genotypes in the VCF files were imputed using LinkImputeR^16^ with the following filters: max missingness of 0.30, minor allele frequency (MAF) of 0.01, minimum depth of 8, and Hardy–Weinberg equilibrium threshold of *p* = 0.0001. The resulting data set had an imputation accuracy of 0.9778 and a correlation value of 0.8764, with 1598 accessions and 68,392 SNPs remaining.

The data set was filtered to only include accessions in the USDA apple germplasm collection that were relevant to modern apple development, which includes accessions labelled as *M. domestica* (*N* = 1154), *M. sieversii* (*N * = 195), *Malus* (L.) *baccata* Borkh. (*N* = 40), *Malus floribunda* Sieb. ex Van Houtte (*N* = 17), *Malus orientalis* Uglitzk. (*N* = 17), and *M. sylvestris* (*N* = 15). Next, the VCF file was converted using PLINK v1.07^17,18^ and filtered for MAF 0.01, resulting in 1438 accessions and 47,925 SNPs.

Our genotype calling pipeline assumes all accessions are diploid (2x), and we, therefore, aimed to exclude triploid accessions (3x). Previous work has confirmed that triploids can be identified from GBS data due to their excessive heterozygosity^19^. We examined heterozygosity by individual, and contrasted these values with labels available in the USDA germplasm database for 2x, 3x and 4x accessions (Supplementary Fig. S4). Using a Tukey test, we determined that accessions labelled as 3x were significantly more heterozygous than 2x (*p* < 1 × 10^−15^) or 4x (*p* = 1.385 × 10^−4^) individuals. There was no significant difference in heterozygosity between 2x and 4x accessions, indicating that the accessions labelled as 4x were likely all autotetraploids and could, therefore, be treated as diploid for the purposes of genotype calling and all downstream analyses. The mean proportion of heterozygous genotypes was 0.191 for accessions labelled as 2x, 0.226 for 3x accessions, and 0.182 for 4x accessions. Based on these results we removed 168 accessions with heterozygosity >0.21 that we inferred to be triploid, including 28 labelled as 2x, 51 labelled as 3x, and 2 labelled as 4x. There were 62 accessions labelled as 3x that were not removed using this filter. The majority of the accessions removed (*N* = 147) were labelled as *M. domestica*. After filtering, 1270 accessions remained.

### Sample curation

To address potential mislabelling of species within the USDA apple germplasm collection, we used the programme fastSTRUCTURE^20^ to evaluate relatedness among samples. First, we included only SNPs anchored to chromosomes 1 to 17, resulting in 46,022 SNPs. Next, we removed accessions that were suspected of being clonally related. To do this, we calculated pairwise identity-by-descent (IBD) using PLINK^17,18^ and only retained one accession per clonal group where proportion IBD (pi-hat) > 0.9, which removed a further 207 accessions. Only SNPs with MAF > 0.01 in the remaining accessions were retained, and these were then pruned for linkage disequilibrium (LD) using the PLINK filter (--indep-pairwise 10 3 0.5), resulting in 27,871 SNPs. The data set of 1063 accessions included samples labelled as *M. domestica* (*N* = 822), *M. sieversii* (*N* = 184), *M. baccata* (*N* = 25), *M. floribunda* (*N* = 2), *M. orientalis* (*N* = 17), and *M. sylvestris* (*N* = 13). We examined values of *K* from 3 to 10 using fastSTRUCTURE and decided on *K* = 3 based on the choose.py function, which determined that three components were needed to explain structure in the data (Supplementary Fig. S5). *K* = 3 resulted in a partition containing primarily *M. domestica*, a partition containing primarily *M. sieversii*, and a partition containing primarily *M. floribunda* and *M. baccata* accessions.

Our aim was to assess the process of domestication and breeding before the use of these wild species during modern cultivar development, and thus, we excluded samples that are likely recent hybrids generated from the use of these wild species during modern breeding. Some samples labelled as *M. sieversii* were suspected to be the result of hybridisation with *M. domestica* cultivars, and we also aimed to exclude these from downstream analyses. To achieve this, we removed from further analysis 67 *M. domestica* accessions with <0.80 assignment probability to the *M. domestica* cluster and 69 *M. sieversii* accessions with <0.80 identity to the *M. sieversii* cluster.

### Relationship between domesticated apples and their progenitors

We returned to the genotype table prior to MAF and LD filtering for fastSTRUCTURE analysis and retained only accessions identified as primarily *M. domestica*, *M. sieversii*, and *M. sylvestris*. Next, we repeated the MAF filter of 0.01 and LD-pruning using PLINK. The curated *Malus* data set contained 883 accessions and 23,006 SNPs.

A principal components analysis (PCA) was conducted with the smartPCA module of the EIGENSOFT package^21^. Equal sample sizes of the ancestral populations (*N* = 13 for *M. sieversii* and *M. sylvestris*) were selected as this has been shown to be a crucial factor in accurately inferring genetic relatedness based on PCA^22^. After establishing the PC axes based on these ancestral populations, the remaining *M. sieversii* and *M. domestica* accessions were projected onto the axes and 13 accessions were identified that were likely mislabelled or hybrids, and these were either re-labelled or removed (Supplementary Fig. S6). We repeated the PCA excluding the mislabelled samples, which included 11 *M. sylvestris*, 115 *M. sieversii* and 749 *M. domestica* and a total of 22,934 SNPs after a MAF filter of 0.01 and LD-pruning as described above. We established PC axes using 11 accessions for *M. sieversii* and *M. sylvestris* and then projected the remaining accessions onto these axes as described previously^23,24^.

In order to detect if gene flow between wild apples (*M. sieversii* and *M. sylvestris*) and domesticated apples differed for cider and dessert apples, we labelled accessions according to primary use based on Migicovsky et al.^25^. We used the label ‘dessert’ to include cultivars labelled as both dessert and cooking apples in the USDA Germplasm Resources Information Network (GRIN) database and other online sources. A Mann–Whitney *U*-test was performed to determine if cider (*N* = 69) and dessert (*N* = 288) apples significantly differed along PC1 and PC2. Finally, we computed F3 statistics using the qp3Pop test in ADMIXTOOLS^26^ to test for introgression from wild species into cider or dessert apples.

### Genome-wide scan for selection in *M. domestica*

To identify genomic regions that may have been targets of positive selection in *M. domestica*, we compared *M. domestica* (*N* = 749) to *M. sieversii* (*N* = 115) using 33,266 SNPs. Genomic regions under positive selection since domestication in *M. domestica* should be significantly differentiated from its primary progenitor, *M. sieversii*. We calculated Fst for each SNP according to Weir and Cockerham^27^ and considered SNPs with Fst values within the top 5% of genome-wide Fst values as potential selection candidates.

Next, we inferred haplotypes using fastPHASE^28^ and calculated the cross population extended haplotype homozgosity (XP-EHH) statistic using the selscan software with the --trunc-ok and --max-gap 1,500,000 options^29^. We contrasted haplotype diversity between *M. sieversii* (the ‘reference’ population) and *M. domestica*. Similar to Fst, we considered selection candidates as those SNPs with XP-EHH values within the top 5% of positive values genome-wide, indicating extreme haplotype homozygosity in *M. domestica*. Genomic regions containing SNPs within the top 5% of both the Fst and the XP-EHH distributions were identified as putatively under selection in *M. domestica*.

Within the genomic regions under putative selection during domestication we conducted a gene ontology enrichment analysis to determine if any GO terms were overrepresented, suggesting possible metabolic or functional changes in the apple genome due to domestication. To conduct the analysis, we obtained the complete gene annotation data set from the Genome Database for Rosaceae that accompanied version GDDH13 v1.1 of the reference genome^12,30^.

We searched for all genes where the entire region fell within +/− 50 kb of a SNP identified as a candidate for selection and reduced the list to unique genes (MD IDs), which resulted in 1738 genes of interest, including 1073 which were associated with at least one GO term. We imported the genome-wide annotations as well as those under putative selection in *M. domestica* into the topGO package in R^31^. We used topGO to test for gene enrichment in biological process ontology using the algorithm = ‘weight01’, which takes GO hierarchy into account, and the statistic = ‘fisher’.

### Relatedness within *M. domestica*

To investigate patterns of relatedness within *M. domestica*, we filtered the genotype table, following removal of triploid accessions, for only accessions labelled as *M. domestica*, regardless of hybridisation. The resulting data set included 1005 *M. domestica* accessions and an additional MAF filter of 0.01 resulted in 31,426 SNPs remaining. We estimated IBD for all pairwise comparisons among the accessions using PLINK^17,18^. Groups of clones were defined as having IBD > 0.90 among all members. We visualised clonal relationships using the ‘network’ package in R^32^.

Next, we reduced the data set to unique cultivars by only including one accession from each clonal group, resulting in the removal of 179 accessions. After filtering for MAF > 0.01, the final data set contained 31,378 SNPs genotyped in 826 accessions. We used the pairwise IBD matrix to identify putative first-degree relationships (i.e., parent–offspring, full sibling, or equivalent) among the *M. domestica* samples. The expected IBD value for first-degree relationships is 0.5. However, observed IBD values for first-degree relatives are expected to vary due to genotyping error, errors in reference genome assembly, and low and uneven SNP density. To calibrate our IBD thresholds for defining first-degree relatives, we identified 55 known parent–offspring pairs from the literature and examined the range of IBD values (Supplementary Table S2)^33,34^. We removed the two lowest values, which were likely due to mislabelled accessions. The remaining IBD values ranged from 0.4158 to 0.5625, and we therefore considered pairs of samples with IBD values greater than or equal to 0.4158 and less than or equal to 0.5625 as putative first-degree relatives. As a result, accessions referred to as first-degree relatives within this manuscript are inferred based on IBD values. Such values do not necessarily reflect first-degree relatives and could be generated from more complex familial relationships due to backcrossing to close relatives and other complex crossing schemes across generations. In addition, some accessions, which represent true first-degree relatives may not fall within this range of IBD values and, therefore, be missed by this analysis. We visualised first-degree relatives using the ‘network’ package in R^32^. Lastly, we reduced the data set to only accessions which had a first-degree relationship with one of the top 9 apple cultivars sold in the USA in 2018^10^. Sequencing for one of the cultivars, ‘Empire’, failed and therefore our analyses were restricted to 8 of the top 9 cultivars, which included ‘Gala’, ‘Red Delicious’, ‘Granny Smith’, ‘Fuji’, ‘Honeycrisp’, ‘Golden Delicious’, ‘McIntosh’, and ‘Pink Lady®’. Visualisations of pedigree relationships were produced using the ‘network’ package in R^32^.

### Genome-wide scan for selection in *M. domestica* during recent breeding

Within *M. domestica*, we examined dessert and cider apples for evidence of selection using both Fst and XP-EHH scans, as performed above for *M. domestica* and *M. sieversii*. SNPs in the top 5% of Fst values were separately tested for overlap with the highest (selection in dessert apples) or lowest (selection in cider apples) 5% of XP-EHH values. Once again, we tested unique genes within +/− 50 kb of a SNP for GO enrichment using the topGO package in R^31^.

Next, we conducted genome-wide association study (GWAS) and XP-EHH scans for traits that may have experienced improvement after domestication. A new reference genome^12^ and new imputation method^16^ were made available following the publication of our previous work and, therefore, our current study includes over three times as many SNPs as the initial GWAS performed in Migicovsky et al.^25^. We therefore retrieved the phenotype data from Migicovsky et al.^25^ and repeated the GWAS for fruit colour (red (*N* = 389) or green (*N* = 131)), fruit firmness (soft (*N* = 278) or firm (*N* = 310)) and fruit size (small (*N* = 320) or large (*N* = 276)) using Tassel v.5.2.48 with the inclusion of a kinship matrix and PCs 1 to 3^35^. For each of these binary phenotypes, we performed an XP-EHH analysis by comparing accessions with one phenotype (e.g., red) to accessions with the other (e.g., green). To verify that the colour GWAS identified only a single significant locus, we used a multi-locus model in the R package ‘mlmm’ v.0.1.1 to perform GWAS^36^ again including the kinship matrix and PCs 1 to 3. MLMM incorporates significant SNPs as cofactors using a stepwise regression. The optimal model was selected using the extended Bayesian information criterion (EBIC) and plotted using the plot_opt_GWAS function^36^. We examined the overlap between the top 5% of XP-EHH SNPs and those passing the Bonferroni-corrected threshold for GWAS. GWAS and XP-EHH results were visualised using the ‘qqman’ R package^37^, with the location of the D5Y SNP (chr3:30698039) in *NAC18.1* (MD03G1222600) indicated on the firmness plot.

The eight cultivars we sequenced that are among the top apples sold in the USA were all homozygous for the desirable firm Y allele at the D5Y SNP in the *NAC18.1* gene. To determine the likelihood of observing homozygosity for this allele across eight random cultivars, we randomly sampled 8 *M. domestica* accessions without replacement 10,000 times and counted the frequency with which all eight accessions were homozygous for the firm allele.

## Results

We collected over 780 billion nucleotides of DNA sequence from the USDA apple germplasm collection, which includes 1949 apple accessions originating from 50 different countries. We used these data to correct and improve the labelling of accessions, and to remove samples unsuitable for downstream analyses. For example, we determined that approximately 13% of the *M. domestica* accessions within the collection were triploid (Supplementary Fig. S4). We also identified numerous instances where accessions were likely the result of recent hybridisation with wild species and/or accessions’ species labels were incorrect (Supplementary Figs. S5 and S6). We implemented several quality filtering steps to ensure that each accession was assigned to the correct species before proceeding with downstream analyses, and each step is described in the Materials and Methods, Table S1 and Supplementary Fig. S2.

### Genomic insights into apple domestication

To examine the effects of domestication on apple genetic diversity, we assessed the genetic contributions of the wild relatives, *M. sieversii* and *M. sylvestris*, to cider and dessert apples. Using the F3 test, cider (f3 = −0.055173) and dessert apples (f3 = −0.042006) had negative f3 values, suggesting that both cider and dessert apples included ancestry from both wild relatives, *M. sieversii* and *M. sylvestris*.

**Fig. 1:**
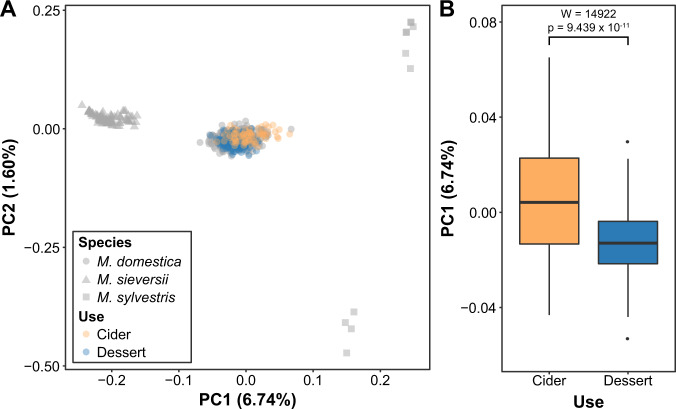
Principal components analysis (PCA) showing population structure for *M. domestica* (*N* = 749), *M. sieversii* (*N* = 115), and *M. sylvestris* (*N* = 11). **A** PC axes were established using equal sample sizes (*N* = 11) of the wild species and the remaining accessions were projected onto the axes. The percent variance explained by each PC is indicated in parentheses. **B** Comparison of PC1 values for cider and dessert apples demonstrates that dessert apples derive more ancestry from *M. sieversii*, while cider apples derive more ancestry from *M. sylvestris*

We investigated the relative contribution of each wild ancestor to cider and dessert apples by performing ancestry deconvolution using PCA. Our analysis identified two distinct groups of *M. sylvestris* along PC2: accessions from Germany (PC2 > 0) and Macedonia (PC2 < 0). By projecting both cider and dessert apples onto the PCs, we found that cider apples were significantly differentiated from dessert apples along PC1 (*W* = 14922, *p* = 9.439 × 10^-11^), which indicates that cider apples derive more ancestry from *M. sylvestris* while dessert apples derive more ancestry from *M. sieversii* (Fig. 1). Cider apples were also significantly different from dessert apples along PC2 (*W* = 14582, *p* = 1.598 × 10^-9^), with cider apples appearing more closely related to German *M. sylvestris* accessions compared to accessions from Macedonia (Supplementary Fig. S7).

**Fig. 2:**
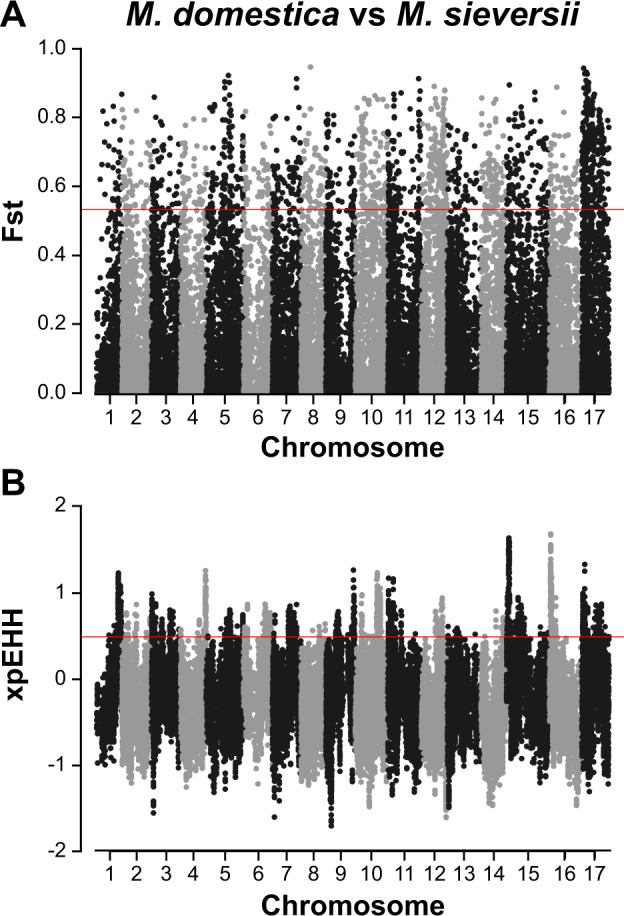
Genome-wide scans for selection during apple domestication. **A** Fst and **B** XP-EHH selection scans comparing *M. domestica* and *M. sieversii*. The horizontal lines indicate the top 5% of values for each test across the genome

To identify regions of the genome that may have experienced selection during domestication, we compared the genomes of *M. domestica* to the genomes of its primary progenitor species, *M. sieversii*, using Fst (Supplementary Table S3 and Fig. 2A) and XP-EHH (Supplementary Table S4 and Fig. 2B). There were 265 SNPs with values within the top 5% of both test statistics (Supplementary Table S5) and we tested for GO enrichment of the genes found within 50 kb of these SNPs (Supplementary Table S6). We report the top ten GO terms for enrichment (Supplementary Table S7), which included ion transport, lipid transport and positive regulation of kinase activity.

### Relatedness among apple cultivars

**Fig. 3:**
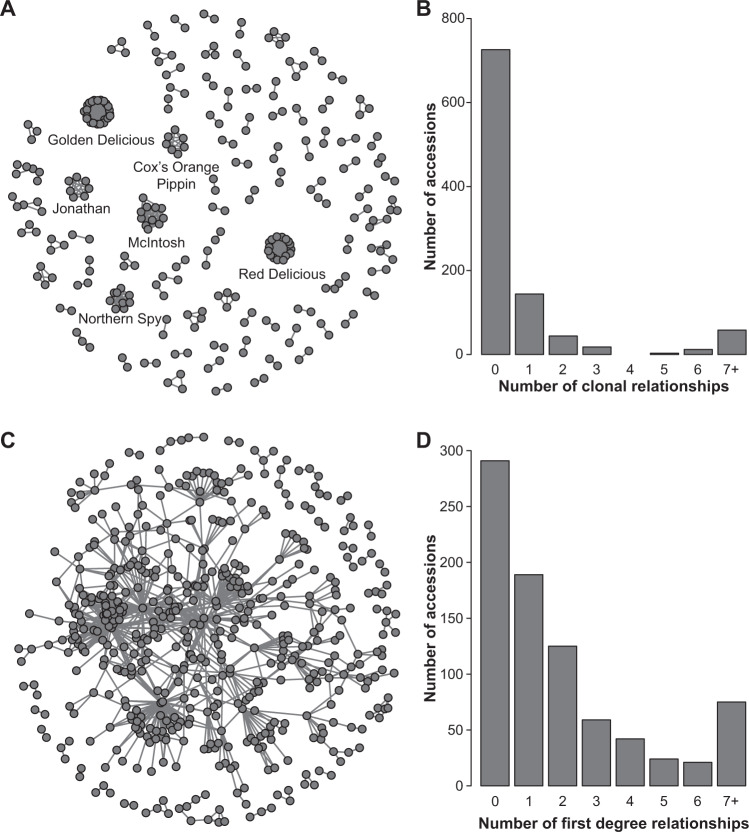
Clonal and first-degree relationships within the USDA apple collection. **A** Network of clonal relationships among accessions labelled as *M. domestica*. Only accessions with at least one clonal relationship (*N* = 279) are included. Each accession is represented by a dot and each line represents a clonal relationship. **B** Number of clonal relationships across entire collection (*N* = 1005). **C** Network of first-degree relationships among apple accessions. Only accessions with at least one first-degree relationship (*N* = 535) are included. Each accession is represented by a dot and each line represents a first-degree relationship. **D** Number of first-degree relationships for each of the unique accessions within the collection (*N* = 826)

By calculating IBD among all pairs of 1005 *M. domestica* accessions, we identified 641 pairs of accessions sharing a clonal relationship. In total, 279 accessions were distributed across 100 clonal groups, with the number of clonal relationships within each group ranging from 1 to 22 (Supplementary Table S8 and Fig. 3A, B). The accessions with the largest number of clonal relationships included ‘Golden Delicious’ (22), ‘Red Delicious’ (15), ‘McIntosh’ (10), and ‘Northern Spy’ (8).

**Fig. 4:**
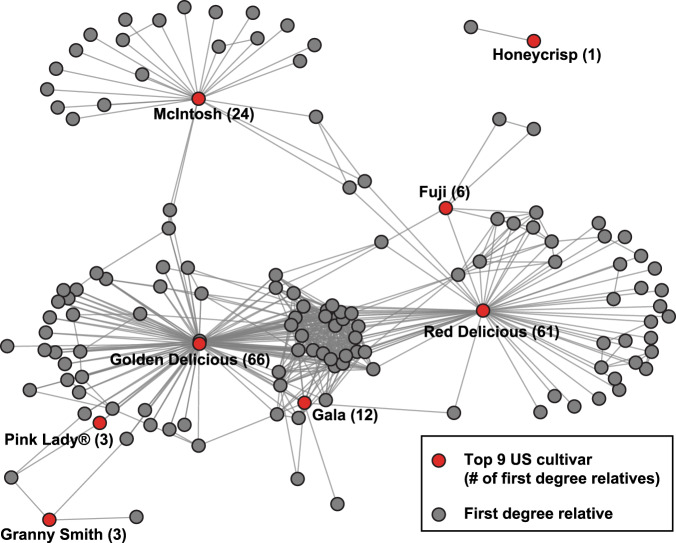
Network of first-degree relationships with the top 8 apple cultivars sold in the United States. In addition to the top apple cultivars, only accessions with at least one first-degree relationship (*N* = 129) are included. Each accession is represented by a dot and each line represents a first-degree relationship

After retaining only one accession from each clonal group, we determined that 535 of the remaining 826 unique accessions had at least one first-degree relative (i.e., sibling, parent–offspring relationship, or equivalent) with another accession in the collection. ‘Golden Delicious’ had the largest number of first-degree relatives (66), while ‘Red Delicious’ had the second largest number of relatives (61). Most accessions (314) had 1 or 2 first-degree relatives, while 44 accessions had >10 (Supplementary Table S9). Over half of the collection (435 out of 826 accessions) were interconnected by a series of first-degree relationships and thus belonged to a single extended pedigree (Fig. 3C, D). When we restricted our analysis to relatives of the top 8 apple cultivars sold in the United States, we identified 129 accessions with a first-degree relationship with at least one of the top 8 apple cultivars (Fig. 4).

### Selection during apple improvement

**Fig. 5:**
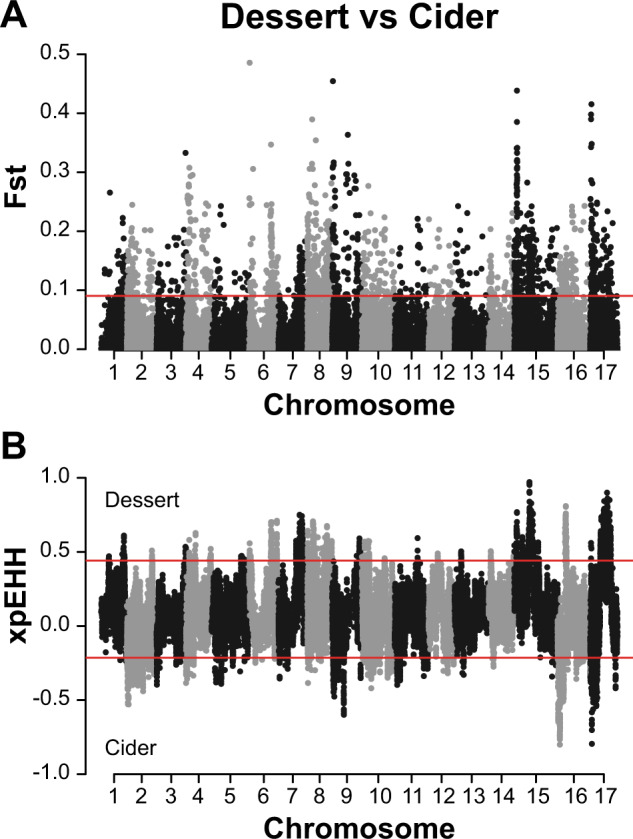
Genome-wide scan for selection during apple improvement. **A** Fst and **B** XP-EHH selection scan profiles comparing dessert and cider accessions. The horizontal lines indicate the top or bottom 5% of values for each test across the entire genome. For XP-EHH, high values indicate selection in dessert apples while low values indicate selection in cider apples

We aimed to identify regions of the apple genome that experienced positive selection due to breeding and improvement. First, we compared dessert and cider apples to identify regions putatively under selection using both Fst (Fig. 5A and Supplementary Table S10) and XP-EHH (Fig. 5B and Supplementary Table S11). Based on the overlap across these two scans, there were 433 SNPs identified as putatively under selection in dessert apples and 81 SNPs in cider apples (Supplementary Table S12). In both instances, we tested for GO enrichment of genes found within 50 kb of these SNPs (Supplementary Table S13) and reported the top ten GO terms (Supplementary Table S14). For dessert apples, the top term was flavonoid biosynthesis while for cider apples, the top terms included malate transport.

**Fig. 6:**
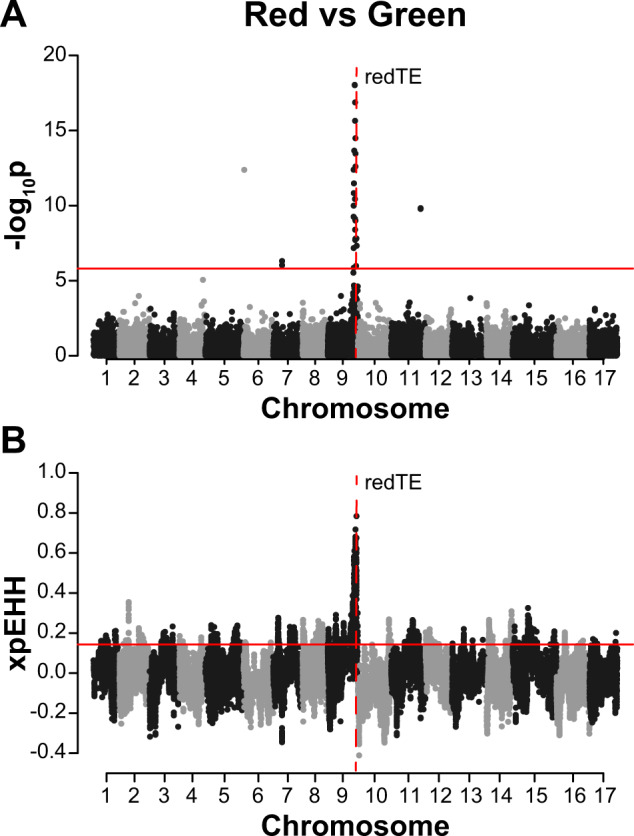
GWAS and XP-EHH selection scan for apple skin colour. Dashed vertical lines indicate the location of the redTE retrotransposon just upstream of the MYB1 gene, a known key regulator of apple skin colour. **A** Manhattan plot of GWAS results for fruit colour. *p*-values are log-transformed and the horizontal line indicates a Bonferroni-corrected *p*-value threshold for significance. **B** XP-EHH selection scan profiles comparing red and green accessions. Positive values indicate selection for red colour. The horizontal lines indicate the top 5% of values across the genome

In addition to the comparison between dessert and cider apples, we conducted GWAS in *M. domestica* accessions for traits likely targeted by breeders and determined if SNPs significantly associated with these traits overlapped with signatures of recent selection as measured by the XP-EHH statistic. We identified a GWAS signal when comparing red vs green apples on chromosome 9 at the *MYB1* gene responsible for fruit colour^38–42^ (Fig. 6A) and found a strong signature of selection at this locus (Fig. 6B). The GWAS resulted in 31 significant SNPs, 22 of which also exhibited extreme positive values of the XP-EHH statistic, indicating intense selection for red skinned apples (Supplementary Table S15). The SNPs with selection signals extended over a 3.43 Mb region on chromosome 9 (chr9:32436474-35868403) and overlapped with the *MYB1* gene (MD09G1278600, chr9:35542733-35549175). We verified that our GWAS signal captures only a single locus of large effect on chromosome 9 and that the SNPs exceeding the significance threshold on other chromosomes were likely mismapped in the reference genome (Supplementary Fig. S8).

Following the same procedure, we tested for selection for increased fruit size. We failed to detect significant SNPs in our GWAS for apple size and did not find any convincing regions of selection for fruit size among SNPs with extreme XP-EHH values (Supplementary Table S16 and Supplementary Fig. S9). For example, the GWAS SNP with the lowest *p*-value on chromosome 13 was located over 350 kb away from a SNP with an extreme XP-EHH value. However, the GWAS SNP with the second lowest *p*-value (chr3:37279998) did exhibit an extreme XP-EHH value indicating a potential signature of selection for large fruit at this locus. This SNP falls within the LEA gene (chr3:37278889-37280205; MD03G1296800), which encodes the late embryogenesis abundant (LEA) protein.

**Fig. 7:**
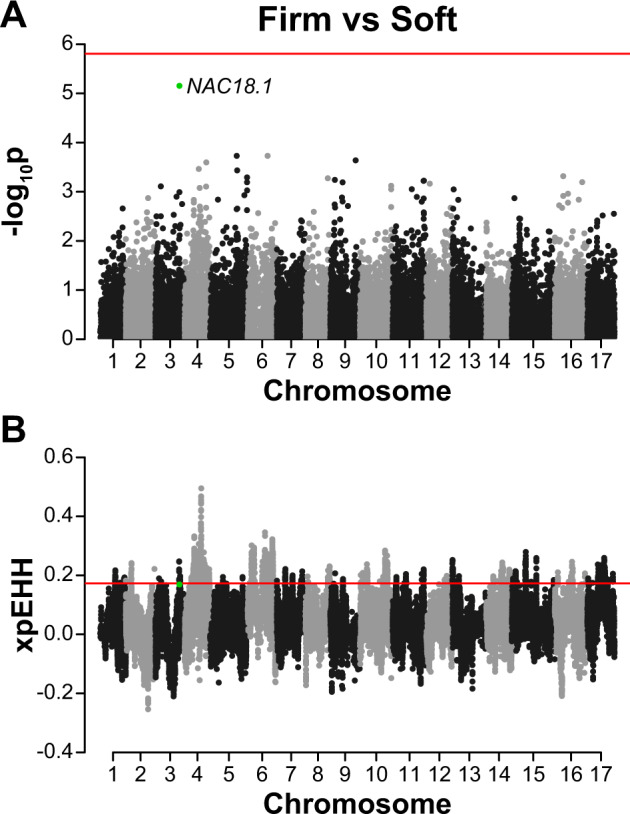
GWAS and XP-EHH selection scan for fruit firmness. The position of a previously identified SNP in *NAC18.1* (D5Y) is indicated with a *green* dot. **A** Manhattan plot of GWAS results for fruit firmness. *p*-values are log-transformed and the horizontal line indicates a Bonferroni-corrected *p*-value threshold for significance. **B** XP-EHH selection scan comparing soft and firm accessions. Positive values indicate selection for firmer apples. The horizontal lines indicate the top 5% of values across the entire genome

We compared soft and firm apples, but did not identify an overlap between the top 5% of XP-EHH values and the region of the genome with the lowest *p*-values from the GWAS (Fig. 7). While no SNPs were found to be significant in the GWAS, the SNP with the lowest *p*-value is located at chr3:30698039 and falls within *NAC18.1*. This SNP, D5Y, results in a nonsynonymous substitution from aspartic acid (D) to tyrosine (Y) at the fifth amino acid of *NAC18.1* and the Y allele has been associated with apple firmness and harvest time in previous studies^25,43,44^. While this putatively causal SNP was not among the 5% most extreme XP-EHH values genome-wide, it was within the top 5.4% of values and thus nearly reached the 5% threshold employed here indicating possible selection for firmer apples (Supplementary Table S17).

We determined that 70.2% of *M. domestica*, 12.2% of *M. sieversii*, and 100% of *M. sylvestris* were homozygous for the firm Y allele. Among the remaining accessions, 23.5% of *M. domestica* and 33.9% of *M. sieversii* were heterozygous, while all other accessions were homozygous for the soft D allele. All of the top 8 apples sold in the United States were homozygous for the desirable Y allele, and we determined that the probability of eight random accessions all being homozygous for the Y allele was 5.7%.

## Discussion

### Insights into apple domestication

There is strong evidence that the apple was domesticated in the forests of Central Asia from the wild progenitor species, *M. sieversii*^4,5,45^. The degree to which there was subsequent hybridisation from other wild species during the several thousand years of its journey around the world remains a topic of intense investigation^6,46–50^. We determined that cider and dessert apples derive ancestry from both *M. sieversii* and the wild European crabapple, *M. sylvestris*. Genomic analyses found support for an Asian origin of the domesticated grape with subsequent introgression from wild species as the grape moved into Europe^51^. Similarly, our results suggest that, as the apple travelled west from its ancestral home in Central Asia, it hybridised with wild European apples. Wild European species have therefore played a crucial role in shaping the genomes of two of the world’s most abundant fruit species.

Using a PCA-based ancestry analysis, we provide evidence that cider apples are generally more closely related to the European crabapple, *M. sylvestris*, than dessert apples are (Fig. 1). Therefore, the genomic contribution of European crabapples is greater for cider apples than for dessert apples. In our study, we also find that cider apples are more closely related to crabapples from Germany compared to those from Macedonia, suggesting that introgression was likely concentrated in northern regions of the European trade routes (Supplementary Fig. S7). Previous work has suggested that the introgression from *M. sylvestris* likely occurred after the cultivated apple was domesticated from *M. sieversii*^5^. Additional work will be required to identify the precise genomic regions that have been introgressed from *M. sylvestris*, and the extent to which *M. sylvestris* contributed to cider apples’ tart and tannic characteristics.

In contrast to our work, Cornille et al.^6^ examined 299 cider and dessert apples, primarily of French origin, and found significantly higher introgression from *M. sylvestris* in dessert apples compared to cider apples. The discrepancy between our findings and those of Cornille et al. may be due to differences in genome coverage and/or sample origin. For example, Cornille et al. relied on 26 microsatellites from primarily French cider apples, while our study made use of >20,000 SNPs from cider apples from a larger geographic range including France (26), the United Kingdom (33), the United States (9), and Sweden (1). Thus, accessions within the USDA germplasm collection represent broader cider apple diversity, and this may explain the differences in our results.

We also compared *M. domestica* to *M. sieversii* to examine the genome for evidence of selection during domestication using Fst and XP-EHH (Fig. 2). We failed to identify specific candidate domestication genes, but we found that putatively selected genomic regions were enriched for genes related to stress response, including target of rapamycin (TOR) signalling^52^, cellular response to phosphate starvation, and cellular response to stress. Recent work by Duan et al.^5^ reported that genes involved in abiotic stress were highly divergent between *M. domestica* and other wild species. This is consistent with our finding that genes involved in stress may have been selected for during apple domestication, potentially to enable adaptation to new environments.

### Relatedness among apple cultivars

Apple is a clonally propagated crop and occasionally a ‘somatic mutant’ or ‘sport’ with a desirable phenotype arises, is clonally propagated, and subsequently commercialised. We found that the accessions with the highest number of sports, or clonal relationships, include many of the most widely sold cultivars in the USA such as ‘Golden Delicious’, ‘Red Delicious’, and ‘McIntosh’ (Fig. 3A)^10^. Among these is the ‘Wijcik McIntosh’, a ‘McIntosh’ sport with a columnar growth habit that makes it desirable for high-density planting^53^. However, the most common apple sports are those with redder fruit since red colour is commercially desirable and is a relatively easy trait to evaluate by eye^54^. For example, red sports of ‘Fuji’, like the accession ‘Fuji Red Sport Type 2’ that we genotyped, are more desirable than the original Fuji, which has poor red colouration^55^.

There are likely two explanations for the extensive clonal relatedness among elite apple cultivars. First, the probability of successful commercialisation of a new sport is likely highest when the desirable phenotype arises on the background of an already commercially successful cultivar. Second, successful cultivars are by definition the most widely planted, and since mutation is random, the probability of observing a sport is highest among the most abundant cultivars. A similar reasoning likely explains the large number of clones of the popular wine grapes ‘Pinot Noir’ and ‘Chardonnay’^51,56^. In total, over 27% of the apples studied here had at least one clonal relationship (Fig. 3B), which suggests that breeders have frequently sought to improve cultivars by incorporating desirable phenotypes discovered initially as sports.

Historically, most of the world’s commercially successful apple cultivars, like ‘McIntosh’^9^, ‘Granny Smith’^57^, ‘Red Delicious’, and ‘Golden Delicious’, were chance seedlings accidentally discovered from uncontrolled pollination events^54^. However, most new cultivars, such as ‘Pink Lady®’, ‘Jazz^TM^’, and ‘Honeycrisp’ were developed by breeders who selected the most desirable trees from among the offspring of bi-parental crosses^54^. We provide evidence that the process of apple breeding involved the repeated use of a limited number of cultivars as parents, resulting in over half of the USDA apple germplasm collection being interconnected by a series of first-degree relationships (Fig. 3C, D). A genetic pedigree analysis of 1400 European apple accessions also revealed extensive relatedness by identifying over one thousand parent–offspring pairs^58^. Significant pedigree relatedness within a germplasm collection was also found in grapes, another clonally propagated perennial crop in which elite cultivars were repeatedly used during breeding^51^. In mango (*Mangifera indica*), most commercially important cultivars are the result of breeding in Florida, and many appear to be closely related to each other^59^. As was the case for clonal relationships, it is the apple cultivars ‘Golden Delicious’ and ‘Red Delicious’, which have the largest number of first-degree relatives: each has >60 first-degree relatives in the collection. Notably, all of the top 8 apple cultivars grown in the USA, with the exception of ‘Honeycrisp’, were interconnected by a series of first-degree relationships (Fig. 4). This means that Americans are eating apples largely from a single family tree.

While ‘Honeycrisp’ is not connected by a first-degree relationship to any of the other elite apple cultivars we genotyped, one of its recorded parents was ‘Honeygold’, an offspring of ‘Golden Delicious’, and the other was ‘Macoun’, an offspring of ‘McIntosh’ (Supplementary Table S9). Thus, presumably the breeder’s intention in performing this cross was to use relatives of two of the most commercially successful apple cultivars in the USA as parents. However, it was discovered that Honeycrisp’s recorded parentage was incorrect: the actual parents of ‘Honeycrisp’ are ‘Keepsake’, an offspring of ‘Northern Spy’, and MN1627, a University of Minnesota selection and offspring of ‘Golden Delicious’, which is no longer available and thus was not genotyped in our study^60,61^. Despite being derived from parents, which were not commercially successful, ‘Honeycrisp’ has achieved widespread commercial success and has been incredibly lucrative for apple growers^10,61^. Since its release in 1991, ‘Honeycrisp’ has been repeatedly used as a parent in breeding, including the release of at least eight commercial cultivars^61^. Perhaps the fact that neither of Honeycrisp’s parents were commercially successful will motivate future apple breeders to explore the diversity that lies beyond the narrow relatedness network we identified here (Fig. 4).

Across the germplasm collection, over 15% of *M. domestica* cultivars had a first-degree relationship with at least one of the top 8 cultivars, indicating the recurrent use of a few cultivars in both breeding and commercial production. It takes about 25 years to develop and release a new apple cultivar^54^, so it is not surprising that apple breeders are likely to invest time and money into making crosses from ‘tried and tested’ cultivars. Using genomics-assisted breeding, however, there may be renewed interest in novel crosses—including the use of wild relatives—when parents and offspring can easily be screened for desirable alleles^62^. From our results, it is clear that breeders have only just begun to tap into the tremendous diversity available in apple and its wild relatives.

### Selection during apple improvement

Among apples grown for commercial production, the primary use is fresh-eating (dessert) although cider apples are grown to be turned into juice and fermented into alcoholic cider. Cider apples are often referred to as ‘spitters’ because they are more tannic and acidic than dessert apples, which are desirable attributes when fermenting apple juice into cider. High conentrations of polyphenols, including flavonoids, increase astringency while malic acid contributes to tartness, both characteristics preferred for cider apples^63,64^. The genomic regions showing signatures of selection in dessert apples were enriched for genes involved in flavonoid biosynthesis, suggesting that there may have been selection in dessert apples for reduced polyphenol content. Signatures of selection in cider apples were identified in genomic regions enriched for genes involved in malate transport, which suggests that enhanced acidity may have been a primary target during cider apple improvement. Overall, these results suggest that genomic regions underlying astringency and tartness are highly differentiated between cider and dessert apples, and this differentiation likely underlies the primary differences in taste profile between these apple types.

In addition to variation in flavour, apples display a wide array of colours that contribute to consumer preference and that are controlled by both environmental and genetic factors. Apple skin colour has been shown to influence consumer preference with redder cultivars deemed to have better eating quality^65^. The long terminal repeat retrotransposon ‘redTE’, upstream of a *MYB1* transcription factor, is a known key regulator of apple skin colour^42^. A homologous *MYB* gene is also associated with fruit colour in date palms (*Phoenix dactylifera*)^66^ and *Citrus*^67^. We identified a single locus associated with skin colour at *MYB1* using GWAS (Fig. 6A and Supplementary Fig. S8). We also found a strong signature of positive selection at this locus, suggesting a rapid increase in the frequency of the ‘redTE’ allele due to selection for red skin during apple improvement (Fig. 6B). Our evidence of selection for a segregating mutation that improves apple redness is consistent with the observation that somatic mutations, leading to redder skin have also been selected for by breeders^54^. An extreme example of this is the ‘Red Delicious’ apple, which originated as a sport from the cultivar ‘Hawkeye’, a chance seedling found in 1872, which was supposedly less than 50% red^68^. ‘Hawkeye’ was renamed ‘Delicious’, and in the nearly 150 years since its discovery, redder and redder sports, including ‘Red Delicious’, have been developed. While the original ‘Delicious’ is rarely grown, over 100 sports with improved colour are now available^68,69^. Our results provide strong genomic evidence that red skin was a key target during recent apple improvement.

In addition to selection for redder fruit, firmness is one of the top priorities for apple breeders^70^ and was likely a key target during apple improvement and breeding. In a study of pear (*Pyrus* spp.), 11 cell wall degradation-related genes were found in selective sweep regions, indicating possible selection for crisp fruit flesh in Asian pears^71^. In apple, the *NAC18.1* gene has been associated with apple firmness in several recent studies^25,43,72,73^. While no SNPs were significantly associated with firmness in our GWAS, the D5Y mutation in *NAC18.1* had the lowest *p-*value of all SNPs tested (Fig. 7). This suggests that we have detected a real association signal, but failed to achieve statistical significance because our GWAS was underpowered and/or our multiple testing correction was too strict. This same SNP was within the top 5.4% of extreme XP-EHH values genome-wide, which suggests that there may have been positive selection for apple firmness.

The weak signal of selection at *NAC18.1* may be due to our observation that the desirable, ‘firm’ allele (Y) is the ancestral allele among angiosperms^25^. However, the undesirable ‘soft’ allele (D) occurs at a much higher frequency within *M. sieversii*, the primary progenitor species of *M. domestica*. Over half (53.9%) of the *M. sieversii* accessions were homozygous for the soft allele in comparison to only 6.28% of the *M. domestica* accessions. Thus, if there was selection for the ancestral firm allele, there may have been enough time for that allele to recombine onto numerous different haplotypes. XP-EHH is intended to detect extended homozygosity around a novel allele on a single haplotype that rapidly rises in frequency immediately after it appears in a population. This test is, therefore, underpowered to detect selection at *NAC18.1*, since the firm allele is ancestral and there have been very few generations of selection. It is worth noting, however, that all of the top 8 cultivars in the USA from the present study are homozygous for the firm allele, which is unlikely to be observed by chance given that the probability of homozygosity for this allele across eight random USDA accessions is 5.7%. Altogether, our results suggest that, by selecting for firmness, apple breeders likely drove the firm *NAC18.1* allele to high frequency.

## Conclusions

Our genome-wide analysis of the USDA apple germplasm collection allowed us to determine the consequences of domestication and subsequent improvement on patterns of apple genetic diversity. By examining population structure among wild species and the domesticated apple, we found that the European crabapple, *M. sylvestris*, likely had a greater genomic contribution to cider apples than to dessert apples. We examined clonal and first-degree relationships, finding widespread use of a few commercially successful cultivars during apple breeding. The use of a small number of ‘elite’ cultivars in apple fails to exploit the immense genomic and phenomic diversity available and leaves the apple industry vulnerable to evolving pests and pathogens and a changing climate. Lastly, we identified evidence of selection for red and firm apples during apple improvement. Ultimately, germplasm collections such as the USDA apple collection described in this study will serve as an essential source of diverse accessions and wild relatives, which have enormous potential for future plant improvement.

### Reporting summary

Further information on research design is available in the Nature Research Reporting Summary linked to this article.

## Supplementary information

Related Manuscript File

Reporting Summary

Supplementary Tables - zipped

Supplementary figures - zipped
