# Supplementary material for: Genomic consequences of apple improvement
Source: Hortic Res. 2021 Jan 1;8:9. doi: 10.1038/s41438-020-00441-7 (PMC7775473; doi:10.1038/s41438-020-00441-7)
Supplement: Supplementary file 4 — Supplementary figures - zipped [file 41438_2020_441_MOESM4_ESM.zip › supp figures/FigureS1.pdf]

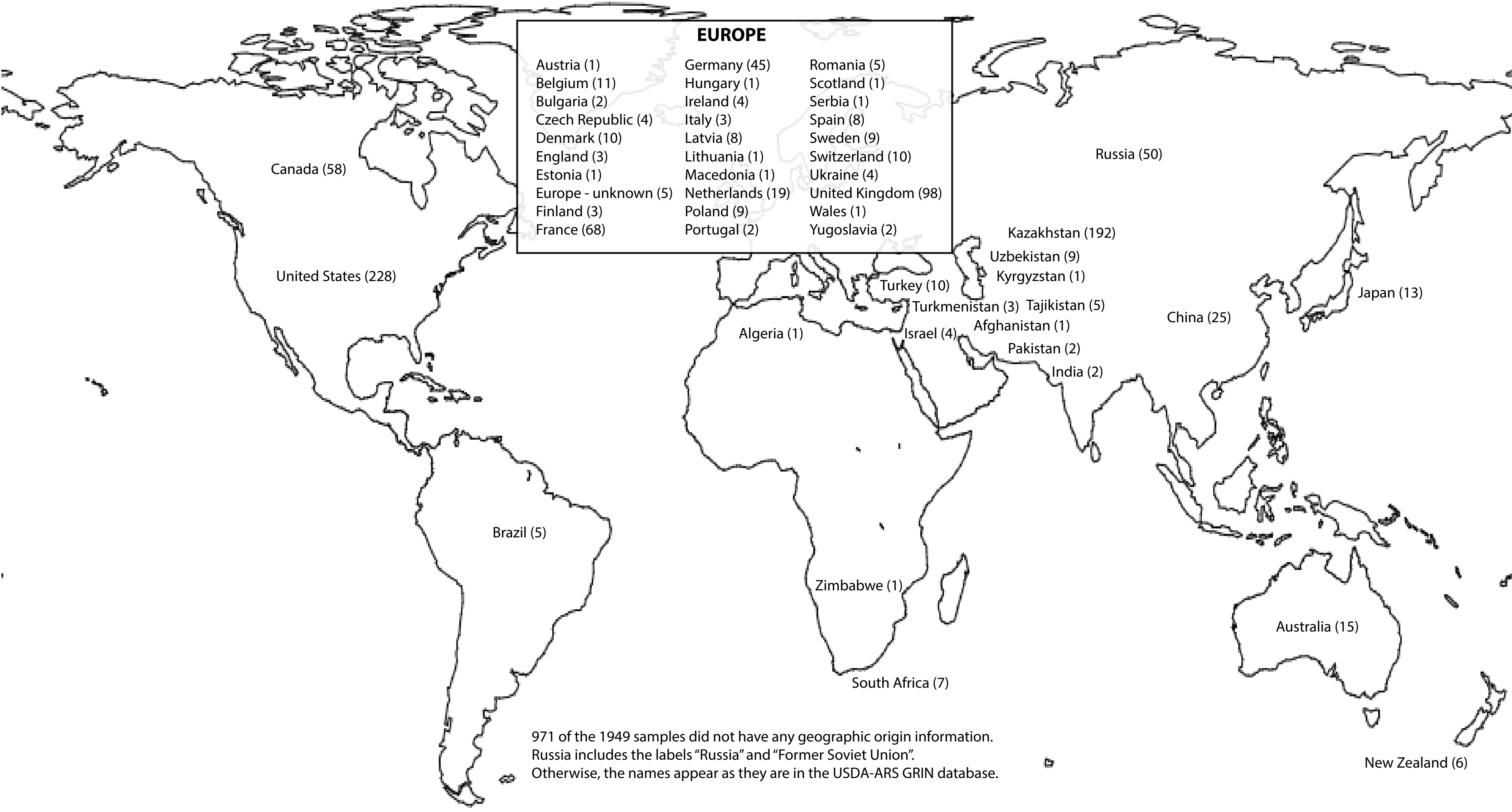

EUROPE
Austria (1)
Belgium (11)
Bulgaria (2)
Czech Republic (4)
Denmark (10)
England (3)
Estonia (1)
Europe - unknown (5)
Finland (3)
France (68)
Germany (45)
Hungary (1)
Ireland (4)
Italy (3)
Latvia (8)
Lithuania (1)
Macedonia (1)
Netherlands (19)
Poland (9)
Portugal (2)
Romania (5)
Scotland (1)
Serbia (1)
Spain (8)
Sweden (9)
Switzerland (10)
Ukraine (4)
United Kingdom (98)
Wales (1)
Yugoslavia (2)
Canada (58)
United States (228)
Brazil (5)
Russia (50)
Kazakhstan (192)
Uzbekistan (9)
Kyrgyzstan (1)
Turkey (10)
Turkmenistan (3)
Tajikistan (5)
Afghanistan (1)
Israel (4)
Pakistan (2)
India (2)
China (25)
Japan (13)
Algeria (1)
Zimbabwe (1)
South Africa (7)
Australia (15)
New Zealand (6)
971 of the 1949 samples did not have any geographic origin information.
Russia includes the labels "Russia" and "Former Soviet Union".
Otherwise, the names appear as they are in the USDA-ARS GRIN database.
