# Supplementary material for: Genomic consequences of apple improvement
Source: Hortic Res. 2021 Jan 1;8:9. doi: 10.1038/s41438-020-00441-7 (PMC7775473; doi:10.1038/s41438-020-00441-7)
Supplement: Supplementary file 4 — Supplementary figures - zipped [file 41438_2020_441_MOESM4_ESM.zip › supp figures/FigureS2.pdf]

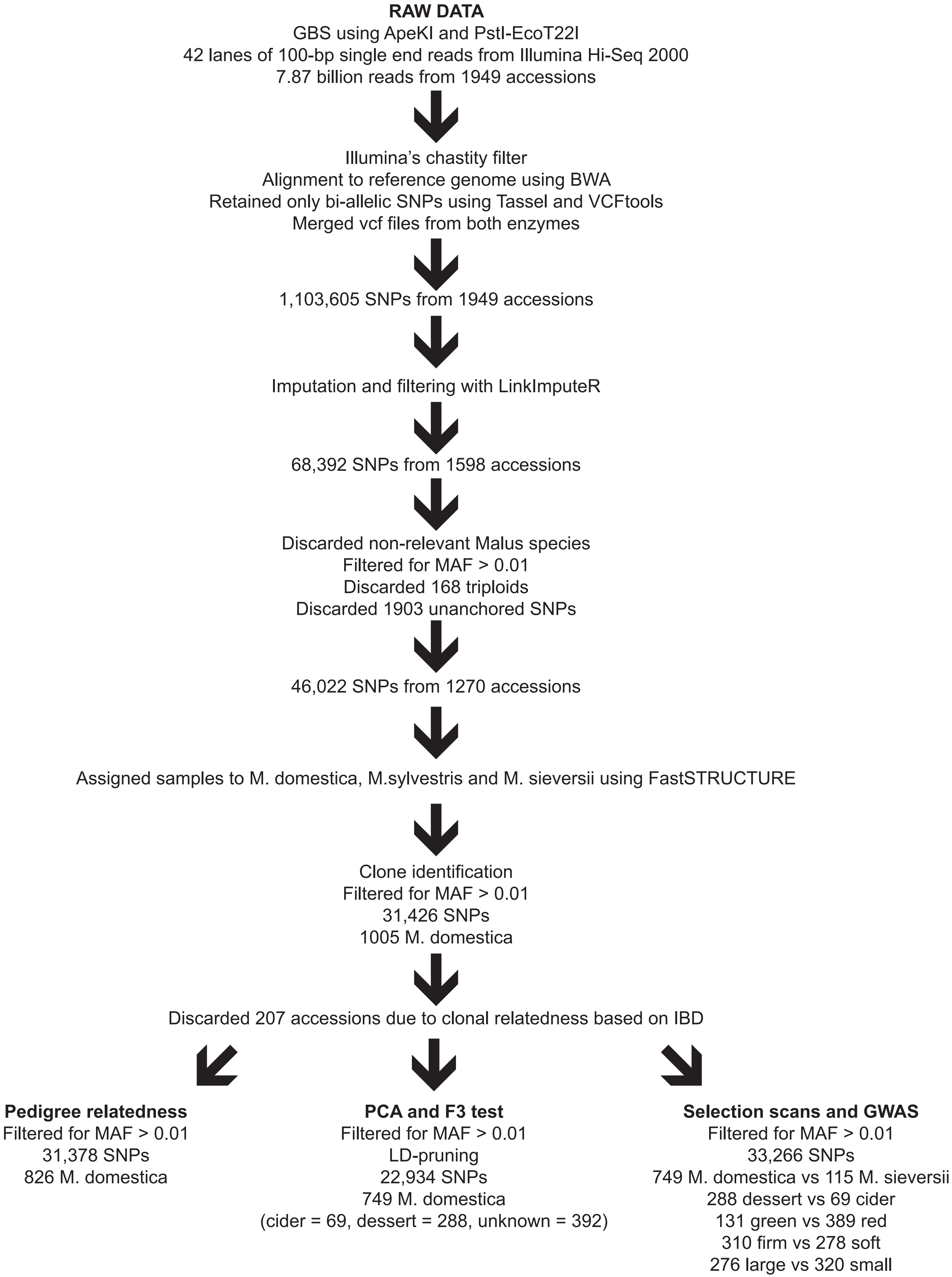
RAW DATA
GBS using ApeKI and PstI-EcoT22I
42 lanes of 100-bp single end reads from Illumina Hi-Seq 2000
7.87 billion reads from 1949 accessions
Illumina's chastity filter
Alignment to reference genome using BWA
Retained only bi-allelic SNPs using Tassel and VCFtools
Merged vcf files from both enzymes
1,103,605 SNPs from 1949 accessions
Imputation and filtering with LinkImputeR
68,392 SNPs from 1598 accessions
Discarded non-relevant Malus species
Filtered for MAF > 0.01
Discarded 168 triploids
Discarded 1903 unanchored SNPs
46,022 SNPs from 1270 accessions
Assigned samples to M. domestica, M.sylvestris and M. sieversii using FastSTRUCTURE
Clone identification
Filtered for MAF > 0.01
31,426 SNPs
1005 M. domestica
Discarded 207 accessions due to clonal relatedness based on IBD
Pedigree relatedness
Filtered for MAF > 0.01
31,378 SNPs
826 M. domestica
PCA and F3 test
Filtered for MAF > 0.01
LD-pruning
22,934 SNPs
749 M. domestica
(cider = 69, dessert = 288, unknown = 392)
Selection scans and GWAS
Filtered for MAF > 0.01
33,266 SNPs
749 M. domestica vs 115 M. sieversii
288 dessert vs 69 cider
131 green vs 389 red
310 firm vs 278 soft
276 large vs 320 small
