# Supplementary figures and images for: Genomic consequences of apple improvement
Source: Hortic Res. 2021 Jan 1;8:9. doi: 10.1038/s41438-020-00441-7 (PMC7775473; doi:10.1038/s41438-020-00441-7)

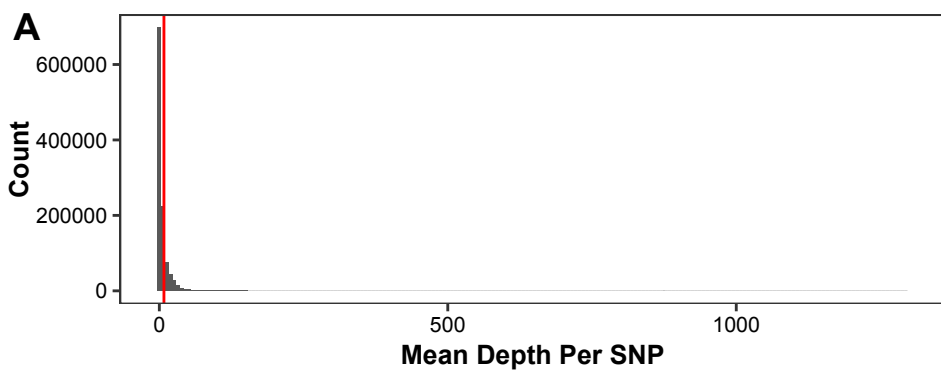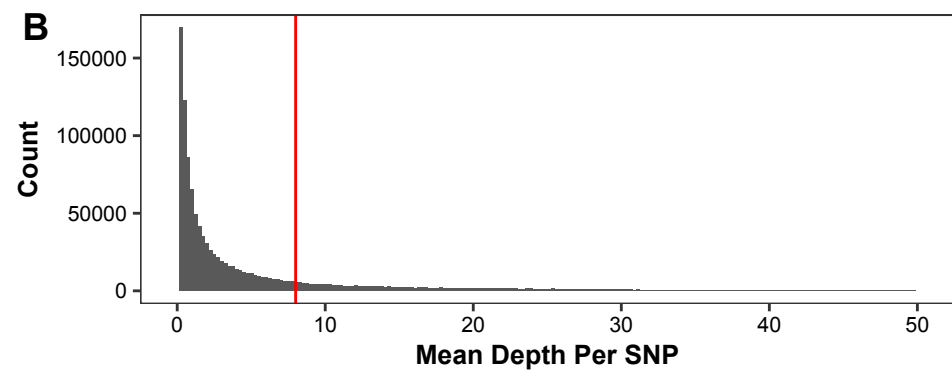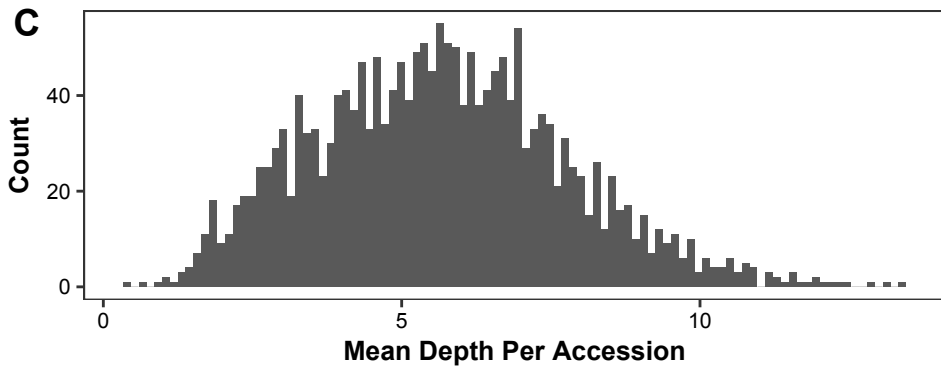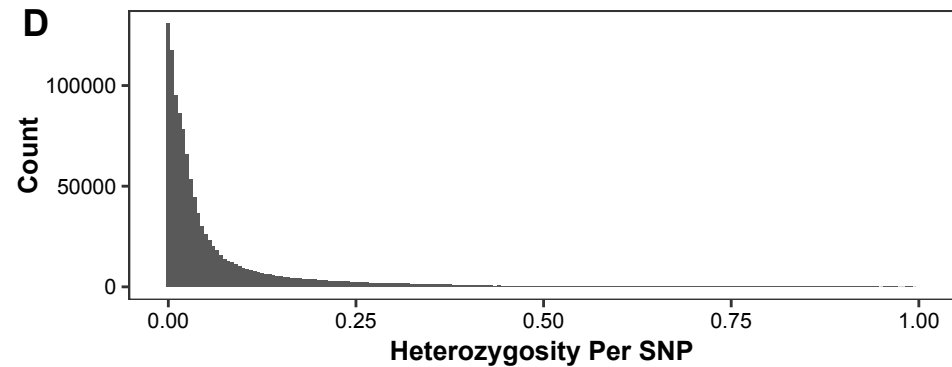

Supplement: Supplementary file 4 — Supplementary figures - zipped [file 41438_2020_441_MOESM4_ESM.zip › supp figures/FigureS3.pdf]

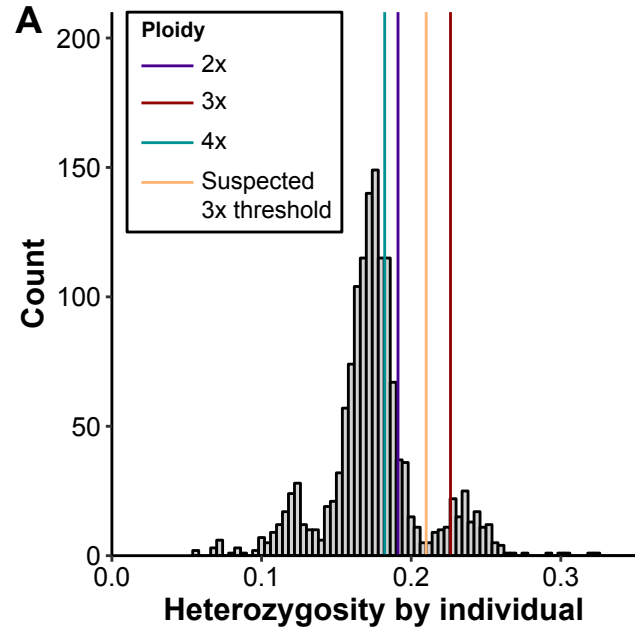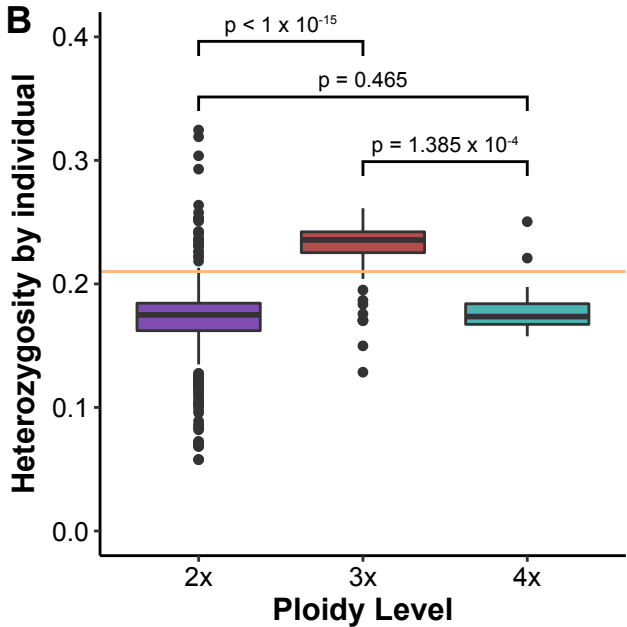

Supplement: Supplementary file 4 — Supplementary figures - zipped [file 41438_2020_441_MOESM4_ESM.zip › supp figures/FigureS4.pdf]

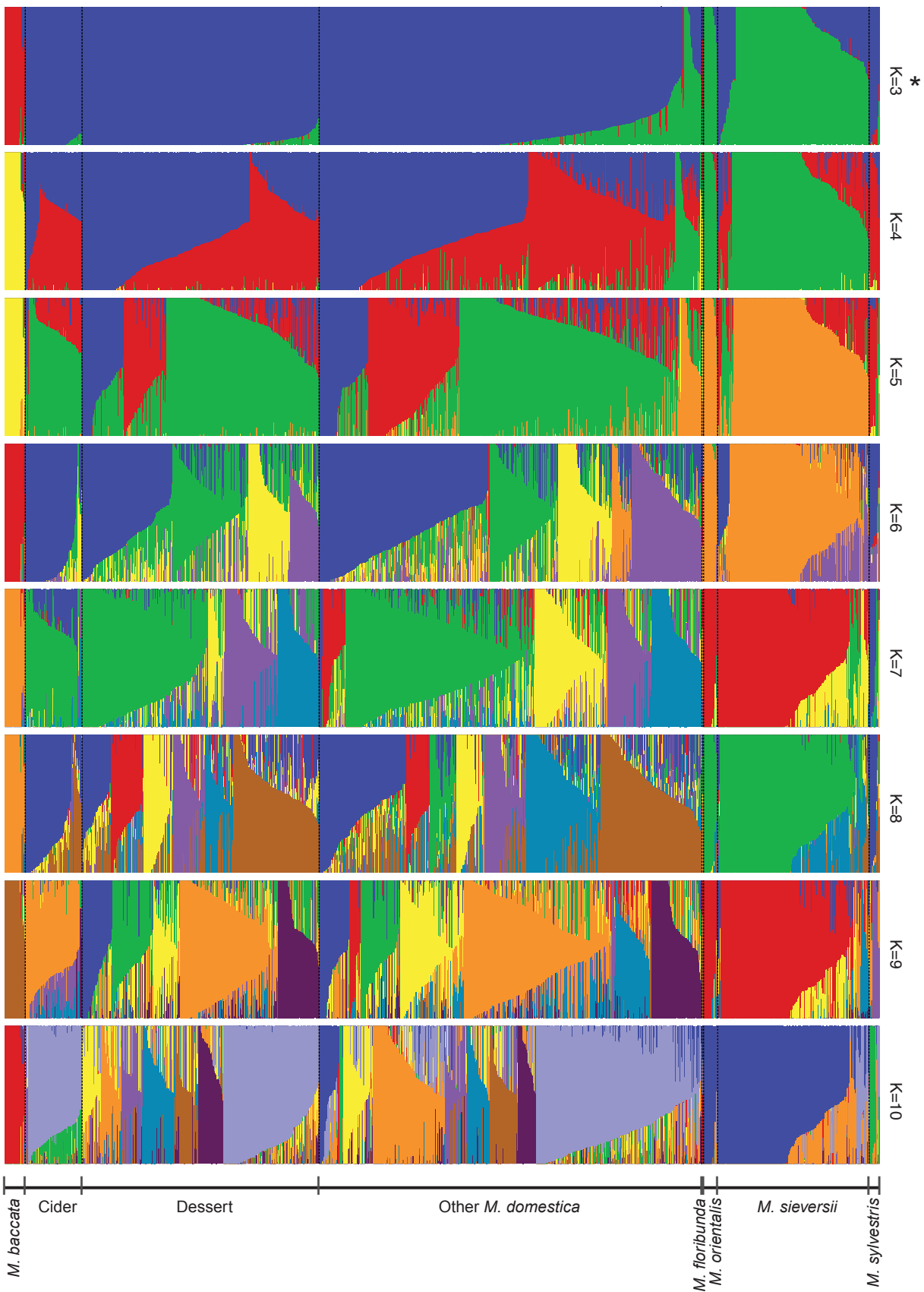

Supplement: Supplementary file 4 — Supplementary figures - zipped [file 41438_2020_441_MOESM4_ESM.zip › supp figures/FigureS5.pdf]

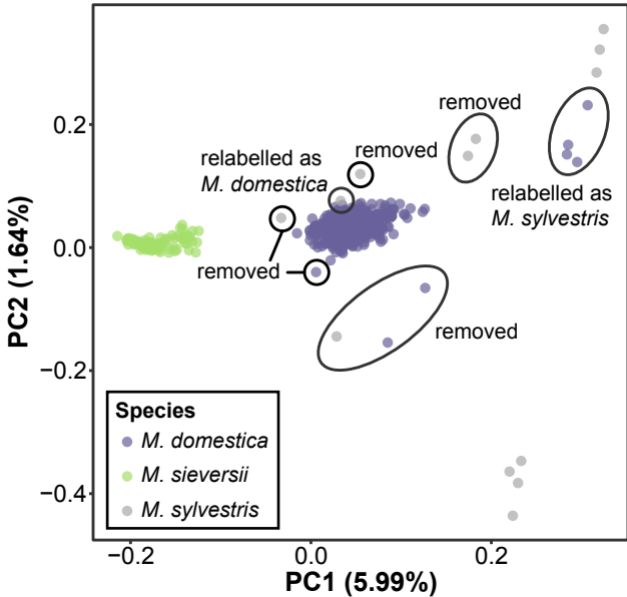

Supplement: Supplementary file 4 — Supplementary figures - zipped [file 41438_2020_441_MOESM4_ESM.zip › supp figures/FigureS6.pdf]

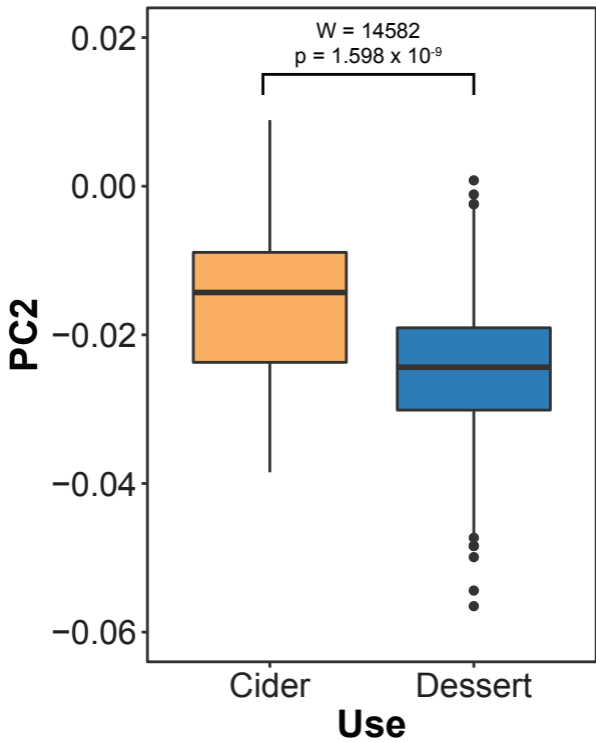

Supplement: Supplementary file 4 — Supplementary figures - zipped [file 41438_2020_441_MOESM4_ESM.zip › supp figures/FigureS7.pdf]

# MLMM GWAS for fruit colour

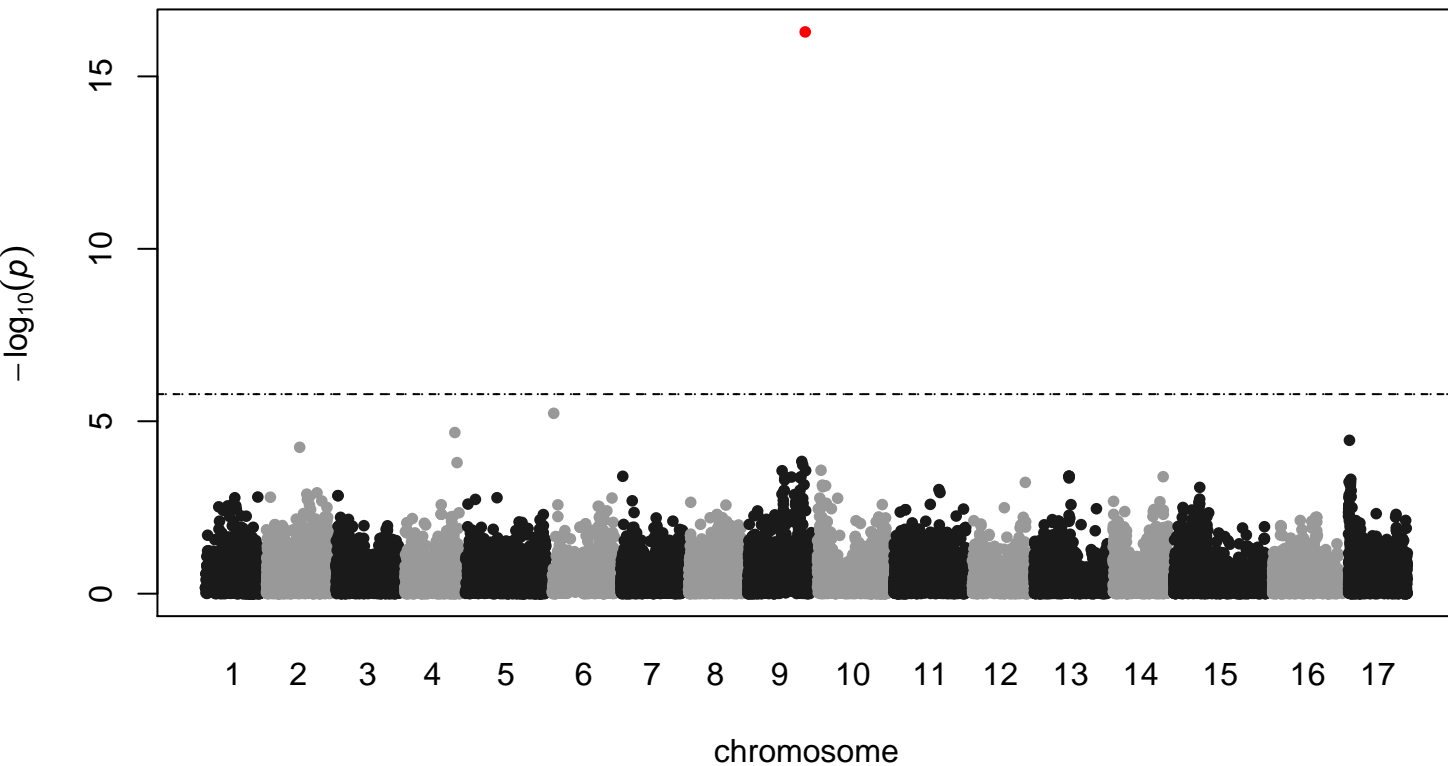

Supplement: Supplementary file 4 — Supplementary figures - zipped [file 41438_2020_441_MOESM4_ESM.zip › supp figures/FigureS8.pdf]

**A**

# Large vs Small

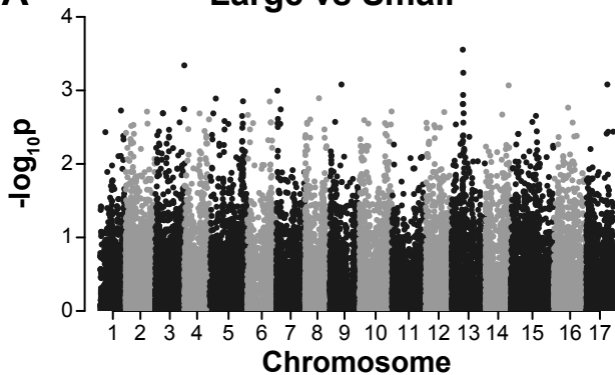**B**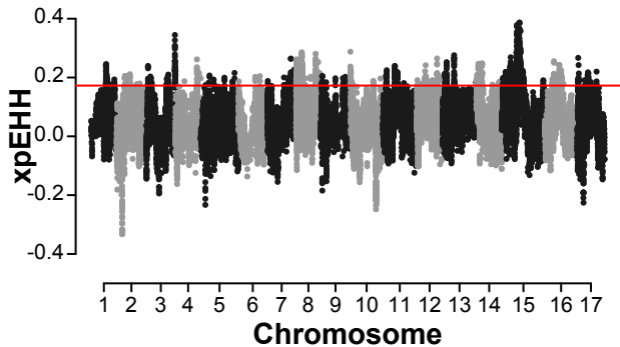

Supplement: Supplementary file 4 — Supplementary figures - zipped [file 41438_2020_441_MOESM4_ESM.zip › supp figures/FigureS9.pdf]
